# Impact of Conventional and Potential New Metal-Based Drugs on Lipid Metabolism in Osteosarcoma MG-63 Cells

**DOI:** 10.3390/ijms242417556

**Published:** 2023-12-16

**Authors:** Daniela S. C. Bispo, Marlene Correia, Tatiana J. Carneiro, Ana S. Martins, Aliana A. N. Reis, Ana L. M. Batista de Carvalho, Maria P. M. Marques, Ana M. Gil

**Affiliations:** 1 Department of Chemistry, CICECO—Aveiro Institute of Materials (CICECO/UA), University of Aveiro, Campus Universitário de Santiago, 3810-193 Aveiro, Portugal; d.bispo@ua.pt (D.S.C.B.); marlene24@ua.pt (M.C.); tatiana.joao@ua.pt (T.J.C.); ascm@ua.pt (A.S.M.); aliananosolini@ua.pt (A.A.N.R.); 2 Unidade de I&D Química-Física Molecular, Department of Chemistry, University of Coimbra, Rua Larga, 300-535 Coimbra, Portugal; almbc@uc.pt (A.L.M.B.d.C.); pmc@ci.uc.pt (M.P.M.M.); 3 Department of Life Sciences, Faculty of Science and Technology, University of Coimbra, Calçada Martim de Freitas, 3000-456 Coimbra, Portugal

**Keywords:** Pt(II) and Pd(II)-complexes, spermine, human osteosarcoma cells, NMR, metabolomics, cellular lipophilic extracts

## Abstract

This work investigated the mechanisms of action of conventional drugs, cisplatin and oxaliplatin, and the potentially less deleterious drug Pd_2_Spermine (Spm) and its Pt(II) analog, against osteosarcoma MG-63 cells, using nuclear-magnetic-resonance metabolomics of the cellular lipidome. The Pt(II) chelates induced different responses, namely regarding polyunsaturated-fatty-acids (increased upon cisplatin), suggesting that cisplatin-treated cells have higher membrane fluidity/permeability, thus facilitating cell entry and justifying higher cytotoxicity. Both conventional drugs significantly increased triglyceride levels, while Pt_2_Spm maintained control levels; this may reflect enhanced apoptotic behavior for conventional drugs, but not for Pt_2_Spm. Compared to Pt_2_Spm, the more cytotoxic Pd_2_Spm (IC_50_ comparable to cisplatin) induced a distinct phospholipids profile, possibly reflecting enhanced de novo biosynthesis to modulate membrane fluidity and drug-accessibility to cells, similarly to cisplatin. However, Pd_2_Spm differed from cisplatin in that cells had equivalent (low) levels of triglycerides as Pt_2_Spm, suggesting the absence/low extent of apoptosis. Our results suggest that Pd_2_Spm acts on MG-63 cells mainly through adaptation of cell membrane fluidity, whereas cisplatin seems to couple a similar effect with typical signs of apoptosis. These results were discussed in articulation with reported polar metabolome adaptations, building on the insight of these drugs’ mechanisms, and particularly of Pd_2_Spm as a possible cisplatin substitute.

## 1. Introduction

Osteosarcoma (OS) is the most common type of primary malignant bone tumor. Although OS can develop at any age, children, teenagers, and young adults (aged from 10 to 30 years) represent the most affected populations [1]. The introduction of chemotherapy for OS treatment has led to a substantial improvement in the prognosis of patients with localized OS (long-term survival rates have increased from less than 20% to 65–70%) [2]. The most effective regimen, designated as MAP, includes the combination of high-dose methotrexate (MTX), doxorubicin (adriamycin, DOX), and cisplatin (*cis*-Pt(NH_3_)_2_Cl_2_, cDDP). However, several side effects and rare toxicities have been identified for this chemotherapeutic protocol [3,4,5], justifying the continuous need for new and more effective anticancer drugs against this type of neoplasia. Since the serendipitous discovery of cDDP as an anticancer agent [6] and its introduction to the clinic [7], this compound has been successfully used in the treatment of several types of cancer (e.g., bone, brain, breast, head, lung and ovarian) [8,9]. However, cDDP has been associated with severe side effects, namely hepatoxicity, nephrotoxicity, neurotoxicity, and ototoxicity, as well as acquired resistance [10,11], thus prompting the search for novel platinum-based anticancer drugs with distinct modes of action and higher efficacy. As a consequence, carboplatin and oxaliplatin (OXA) have been approved for clinical use as second-generation Pt(II)-based metal complexes [8,12], even though they are still associated with some severe deleterious side effects [8,9,13].

Palladium-based compounds have attracted increasing interest as promising alternatives to platinum drugs, due to the chemical similarity between Pd(II) and Pt(II) ions [14]. In addition, given the ability of linear polyaminic ligands to form stable complexes with both metal ions, polynuclear Pt(II) and Pd(II) chelates with polyamines have been the object of intense research in the last two decades [14,15,16,17,18]. Indeed, biogenic amines such as spermine (H_2_N(CH_2_)_3_NH(CH_2_)_4_NH(CH_2_)_3_NH_2_, Spm) have been used as effective coordinating ligands [14], able to bind to more than one metal center and form highly flexible complexes, which allow the formation of long-range intra- and inter-strand crosslinks with DNA. This seems to lead to DNA damage that is more severe and less repairable than for the mononuclear conventional drugs (e.g., cDDP, carboplatin, or OXA) [14]. In particular, the dinuclear Pt(II) and Pd(II) chelates, Pt_2_Cl_4_Spm and Pd_2_Cl_4_Spm (abbreviated to Pt_2_Spm and Pd_2_Spm, respectively), have exhibited favorable in vitro antitumor activity toward different cancer types, including OS [16,17,18], and Pd_2_Spm has been shown to be more effective than its Pt(II) analog in some cases [18]. Regarding OS, Pd_2_Spm exhibited an IC_50_ of about 10-fold lower than Pt_2_Spm [17].

Untargeted nuclear magnetic resonance (NMR) metabolomics has been applied to evaluate the metabolic impact of cDDP on several cancer cell lines, namely lung [19,20,21], brain [22,23,24], breast [25,26,27,28], ovarian [29] and OS [16,17,30,31]. Some of these studies analyzed whole or lysed cells, while others have focused on cellular extracts [18,23,24,28,29] and media [29]. As for OXA, to the best of the authors’ knowledge, it has been assessed by NMR metabolomics of hydrophilic extracts of hepatocellular carcinoma [32] and OS [17] cells. Regarding the new dinuclear Spm chelates, the effect of Pd_2_Spm has been investigated in OS, through high-resolution magic angle spinning (HRMAS) NMR of lysed cells [16] and NMR of hydrophilic extracts [18], having been shown to induce less significant metabolic changes than cDDP, along with comparatively higher cytotoxicity [17]. Concerning the metabolic impact of Pt_2_Spm, a single previous study by the authors has been reported on hydrophilic extracts of a human OS cell line (MG-63) [18]. Although mass spectrometry (MS) is more commonly employed in lipid profiling studies, NMR spectroscopy presents itself as a valuable tool, as it enables the simultaneous observation of several lipid families while also being non-destructive, rapid, and of high reproducibility [33]. In fact, an NMR-based strategy has been previously applied to evaluate the metabolic impact of cDDP on lipophilic extracts of brain [24] and ovarian [29] cancer cell lines. In the former, the evaluation of several brain tumor cell lines with different sensitivities to cDDP treatment showed increased levels of saturated and unsaturated lipids in cDDP-responsive cells, possibly associated with lipid droplet accumulation resulting from cellular membrane degradation or de novo lipid biosynthesis [24]. As for ovarian cancer cell lines exposed to cDDP, an increase in cholesterol, triglycerides (TGs), and polyunsaturated fatty acids (PUFAs) was reported [29], probably reflecting ongoing membrane degradation during apoptotic processes. Moreover, ^1^H HRMAS NMR metabolomics of lysed MG-63 cells have highlighted significant changes in the lipid metabolism upon exposure to either cDDP [16,30,31] or Pd_2_Spm [16], suggesting that lipophilic extracts may provide further information on lipidic metabolic response.

**Figure 1:**
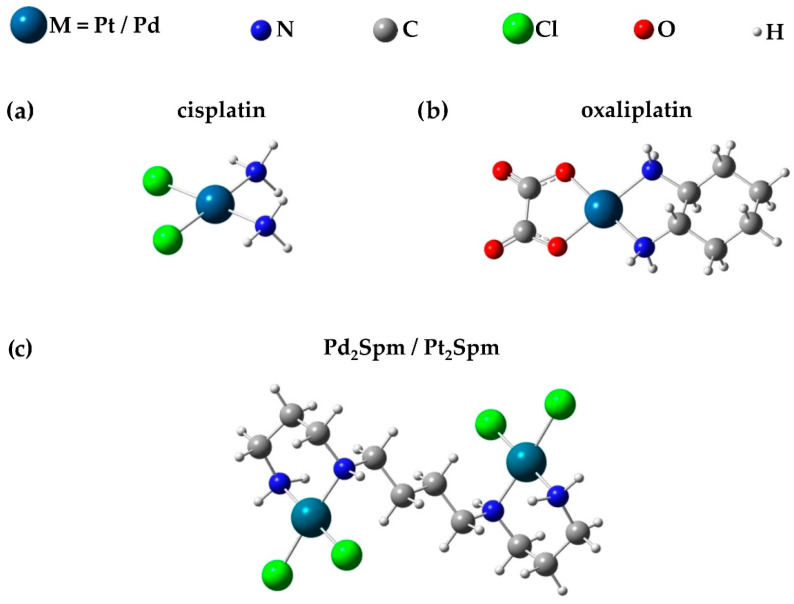
Chemical structures of the Pt(II)/Pd(II) chelates named (**a**) cisplatin (cDDP), (**b**) oxaliplatin (OXA), and (**c**) Pt(II)/Pd(II) Spermine (Spm) (Pt_2_Spm/Pd_2_Spm). The 3D structures shown have been calculated according to reference [34].

In this study, an untargeted NMR metabolomics strategy was followed for the first time to the authors´ knowledge, in order to evaluate the impact of the dinuclear Pt(II) and Pd(II) chelates Pt_2_Spm and Pd_2_Spm on the lipophilic metabolome of a human OS cell line (MG-63), and compare it with the effect of conventional mononuclear Pt(II) drugs cDDP and OXA (chemical structures found in Figure 1). This work builds upon a previous report on the impact of these Pt- and Pd-chelates on the hydrophilic metabolome of the same OS cells [18]. Our results should contribute to a better understanding of the mode of action of these metal-based drugs and unveil potential metabolic markers of OS response to therapy.

## 2. Results and Discussion

### 2.1. NMR Spectra of Lipidic Extracts of OS Cells Exposed to the Different Pt(II)/Pd(II) Drugs

**Figure 2:**
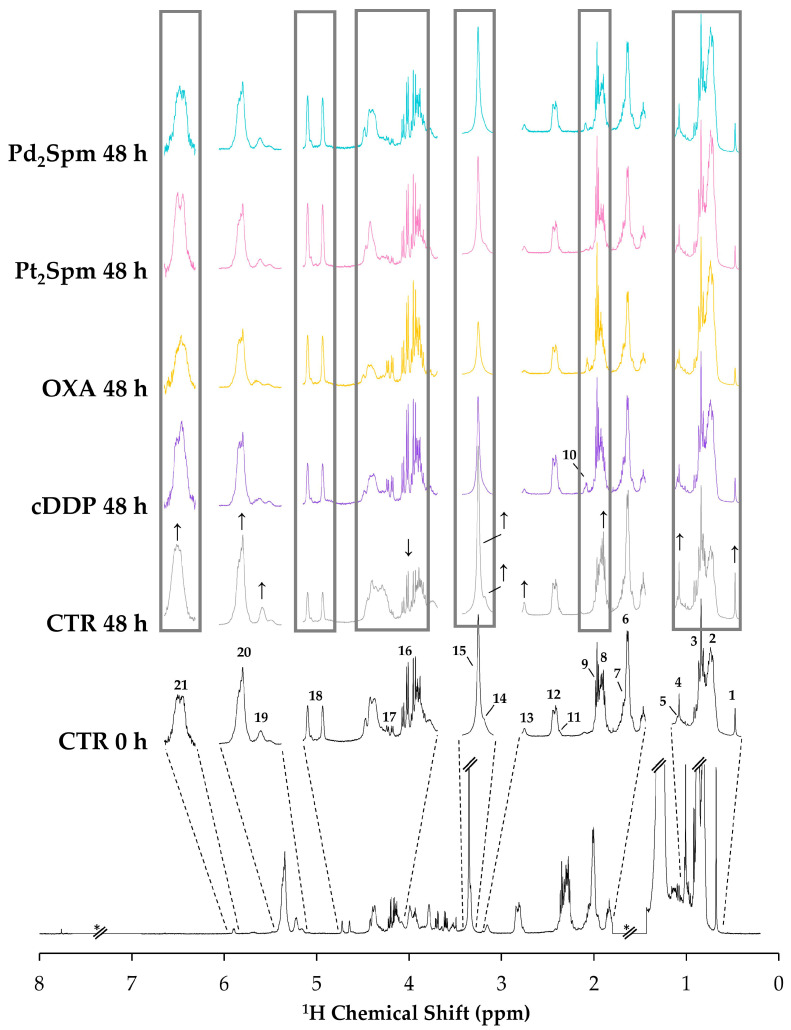
Average normalized ^1^H NMR spectra (500 MHz, CDCl_3_) of lipidic extracts from MG-63 cells prior to exposure (controls, CTR, at 0 h, black trace), following growth during 48 h (CTR at 48 h, grey trace), and upon exposure to 30 μM cisplatin (cDDP 48 h, purple trace), 100 μM oxaliplatin (OXA 48 h, yellow trace), 240 μM Pt(II)-spermine chelate (Pt_2_Spm 48 h, pink trace) and 24 μM Pd(II)-spermine chelate (Pd_2_Spm 48 h, turquoise trace); the drug doses used correspond to their IC_50_ values at 48 h. Apparent peak variations in controls after 48 h are indicated by ↑ or ↓ corresponding to increases and decreases, respectively, whereas the grey rectangles guide the eye to regions where profile changes occur depending on the drug. Peak assignment and multiplicity (see abbreviations at the end of caption): 1. TC (s, 18-CH_3_), 2. Uδ0.80 (br), 3. All FAs except ω-3 (t, CH_3_), 4. FC (s, 19-CH_3_), 5. EC (s, 19-CH_3_), 6. MUFAs (m, -CH_2_-CH_2_-CH=), 7. ω-3 + ω-6 FAs (m, -CH_2_-CH_2_-CH=), 8. FAs in TGs + GPL + EC (m, -CH_2_-CO), 9. FFAs + FAs in 1-MGs (t, -CH_2_-CO), 10. Uδ2.47 (d), 11. LA (t, =CH-CH_2_-CH=), 12. PUFAs (m, =CH-CH_2_-CH=), 13. PtdEtn (br, N-CH_2_), 14. SMs (s, -N (CH_3_)_3_^+^), 15. PtdCho (s, -N(CH_3_)_3_^+^), 16. 1-MGs (ddd, 1-CH_2_ glycerol), 17. TGs (dd, 1-CH_2_/3-CH_2_ glycerol), 18. Uδ4.64 (d); 19. GPLs (m, 2-CH glycerol), 20. UFAs (m, -HC=CH-), 21. Pls (d, O-CH=CH). All numbered peaks (part of lipid spin systems) were chosen for subsequent integration, and a full list of assignments can be seen in Table S1. Abbreviations: 1-MGs, 1-monoacylglycerides; EC, esterified cholesterol; FAs, fatty acids; FC, free cholesterol; FFAs, free fatty acids; GPLs, glycerophospholipids; MUFAs, monounsaturated fatty acids; Pls, plasmalogens; PtdCho, phosphatidylcholine; PtdEtn, phosphatidylethanolamine; PUFAs, polyunsaturated fatty acids; SMs, sphingomyelins; TC, total cholesterol; TGs, triglycerides; UFAs, unsaturated fatty acids; Uδ (m): unassigned signal at chemical shift δ; *, excluded spectral regions; s: singlet, br: broad, t: triplet, m: multiplet, d: doublet, dd: doublet of doublets, ddd: doublet of doublets of doublets.

Figure 2 (bottom trace, in black) shows the average ^1^H NMR spectrum of the lipophilic extracts of control MG-63 cells at 0 h (CTR 0 h), where vertical expansions reveal particular quantifiable resonances arising from: total cholesterol (TC, peak 1), unspecified methyls from all fatty acids (FAs) (peak 2), methyls from FAs except ω-3 FAs (peak 3), free cholesterol (FC) 19-CH_3_ (peak 4), esterified cholesterol (EC) 19-CH_3_ (peak 5), monounsaturated FAs (MUFAs) (peak 6), ω-3 + ω-6 FAs (peak 7), overlapped contributions from FAs in TGs, glycerophospholipids (GPLs) and EC (peak 8), overlapped contributions from free FAs (FFAs) and FAs in 1-monoglycerides (1-MGs) (peak 9), unassigned resonance at δ 2.47 (peak 10), linoleic acid (LA) (peak 11), PUFAs (peak 12), phosphatidylethanolamine (PtdEtn) (peak 13), sphingomyelins (SMs) (peak 14), phosphatidylcholine (PtdCho) (peak 15), 1-MGs (peak 16), TGs (peak 17), unassigned resonance at δ 4.64 (peak 18), GPLs (peak 19), UFAs (peak 20) and plasmalogens (Pls) (peak 21). The ^1^H NMR spectra of MG-63 lipid extracts are dominated by resonances arising from FAs, PtdCho, and UFAs, and a full list of assigned spin systems may be found in Table S1. This set of assignments is broadly consistent with those based on previous ^1^H HRMAS NMR reports of lysed MG-63 cells [16,30,31] and of extracts of MG-63 cells (grown in monolayer and as spheroids) [35,36,37].

Visual inspection of the average normalized spectra of MG-63 cells under different conditions (Figure 2) firstly reveals changes in controls, from 0 to 48 h (Figure 2, black and grey traces), namely relative increases in TC (peak 1) and EC (peaks 4 and 8), FAs in TGs/GPLs (peak 8) PtdEtn (peak 13), SMs (shoulder 14), PtdCho (peak 15), GPLs (peak 19), UFAs (peaks 20) and Pls (peaks 21); and decrease in 1-MGs (peaks 16). These differences are apparent visual changes that seem to accompany tumor cell proliferation alone, however, they require validation through statistical analysis (as shown below). Other apparent profile changes are noted across the spectra for MG-63 cells exposed to the different drugs for 48 h (Figure 2, grey rectangles), including two still unassigned but prominent features numbered as 10 and 18. The relevance and meaning of visual spectral changes upon proliferation (controls alone) and treatment with each Pt(II)/Pd(II) drug are discussed below.

### 2.2. Multivariate and Univariate Statistical Analysis of NMR Data

#### 2.2.1. Multivariate Models of Lipidic Extracts of MG-63 Cells after 48 h of Exposure Time to Pt(II)/Pd(II) Drugs

**Figure 3:**
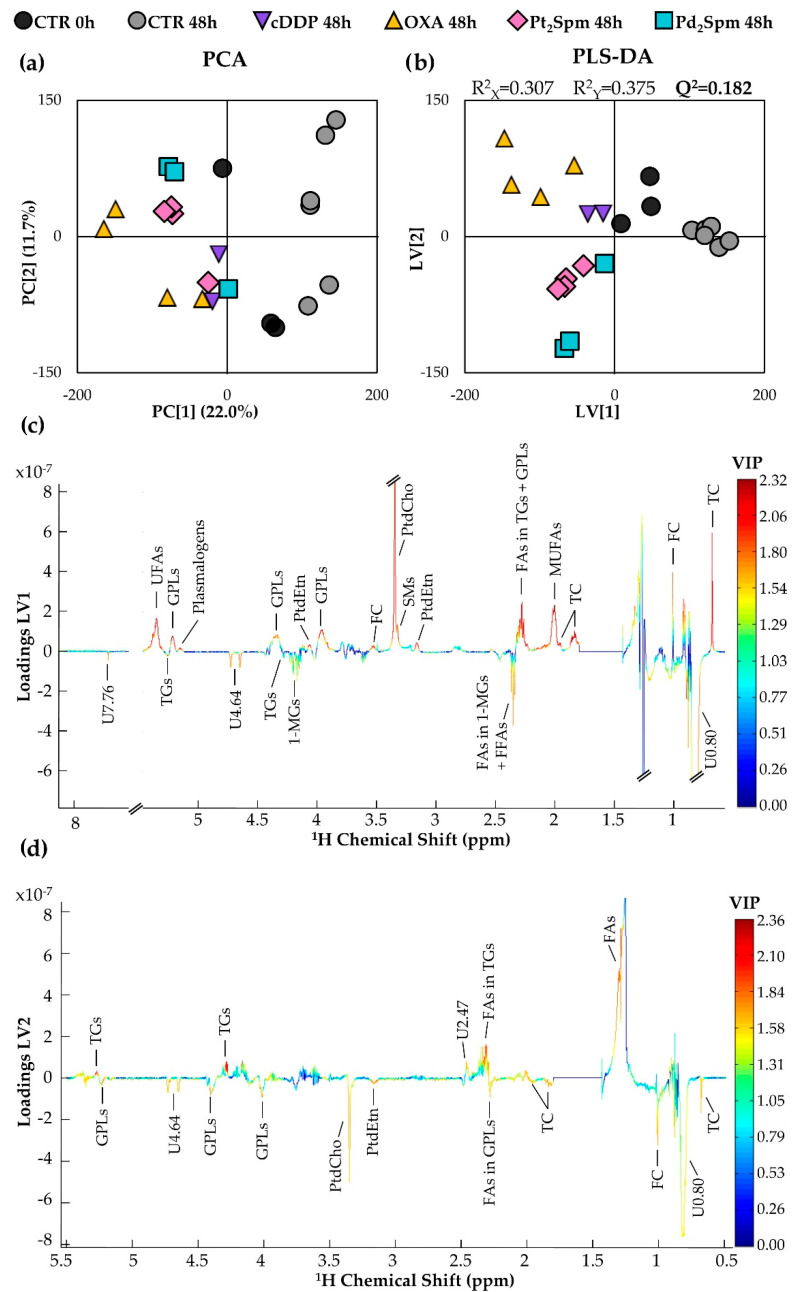
Multivariate statistical analysis of ^1^H NMR spectra of lipid extracts of MG-63 cells (in CDCl_3_) at 48 h of exposure time to cDDP (purple symbols, n = 2, 30 μM), OXA (yellow, n = 4, 100 μM), Pt_2_Spm (pink, n = 4, 240 μM), and Pd_2_Spm (turquoise, n = 3, 24 μM), compared to the controls at 0 h (black, n = 3) and 48 h (grey, n = 6). (**a**) Principal component analysis (PCA) and (**b**) partial least square-discriminant analysis (PLS-DA) scores scatter plots; (**c**) LV1 and (**d**) LV2 loadings plots corresponding to the PLS-DA model shown in (**b**).

Principal component analysis (PCA) and partial-least-squares discriminant analysis (PLS-DA) scores scatter plots obtained with all the spectra (Figure 3a and b, respectively) indicate that controls evolve from 0 to 48 h toward more positive PC1/LV1, whereas all drug-exposed MG-63 cells are placed in negative PC1/LV1. This confirms that controls alone express distinct changes in lipophilic profile with culture time, which should characterize tumor cell proliferation under untreated conditions. Regarding drug-exposed groups, their position in negative PC1/LV1 (Figure 3a,b) probably reflects the less proliferative nature of treated cells. Therefore, sample position along PC1/LV1 seems to provide a qualitative indication of relative MG-63 cell proliferation extension, with more positive LV1 suggesting that higher proliferation rates seem to be associated with higher levels of TC, FC, MUFAs, phospholipids (PtdCho, SMs, PtdEtn, GPLs, and Pls) and UFAs (positive red peaks in Figure 3c), along with possible lower levels of TGs and 1-MGs, along with several unassigned resonances (negative red peaks in loadings plot, Figure 3c). Although no clear group separation is observed for the different drug-exposed groups in the full PCA (Figure 3a), in the corresponding PLS-DA some separation is observed between cDDP- and OXA-treated cells (Figure 3b, purple and yellow symbols, positive LV2) and both Pt(II)/Pd(II)Spm complexes (Figure 3b, pink and turquoise symbols, negative LV2). It is possible, therefore, that spermine complexes induce a partially similar lipid profile, due to the presence of the common ligand. In this case, the corresponding PLS-DA loadings (LV2, Figure 3d) do not show as many resonances with high variable importance to projection (VIP) (suggesting fainter lipid changes between drug groups), but suggest that cells treated with spermine-containing complexes (either Pt_2_Spm or Pd_2_Spm, positioned in negative LV2, Figure 3b) may contain fewer TGs (positive red peaks in LV2 loadings, Figure 3d) and a tendency for higher levels of cholesterol, GPLs, PtdCho and PtdEtn (negative orange/yellow peaks in LV2 loadings, Figure 3d). 

The above suggestions based solely on multivariate analysis need validation through spectral inspection, peak integration, and univariate statistical analysis, as apparently high VIP spectral changes in loadings may not necessarily reflect meaningful statistical relevance. In any case, pairwise PCA (Figure S1a–d) clearly shows a distinction between each of the drug groups compared to the controls (all at 48 h), with the two spermine-containing complexes Pt_2_Spm and Pd_2_Spm exhibiting only partial separation (Figure S1e). In addition, it is interesting to note that PCA of the spectra corresponding to cells exposed to each of the three Pt(II) chelates, cDDP, OXA, and Pt_2_Spm (Figure S1f), indicates a clear separation between OXA (yellow symbols) and Pt_2_Spm (pink symbols), whereas the two cDDP-exposed samples are positioned between those groups. Hence, it is clear that the different ligands in Pt(II) chelates induce distinct lipid profiles reflective of distinct mechanisms of action toward MG-63 cells. 

#### 2.2.2. Lipidic Changes in Untreated Proliferating MG-63 Cells (0–48 h Period)

**Figure 4:**
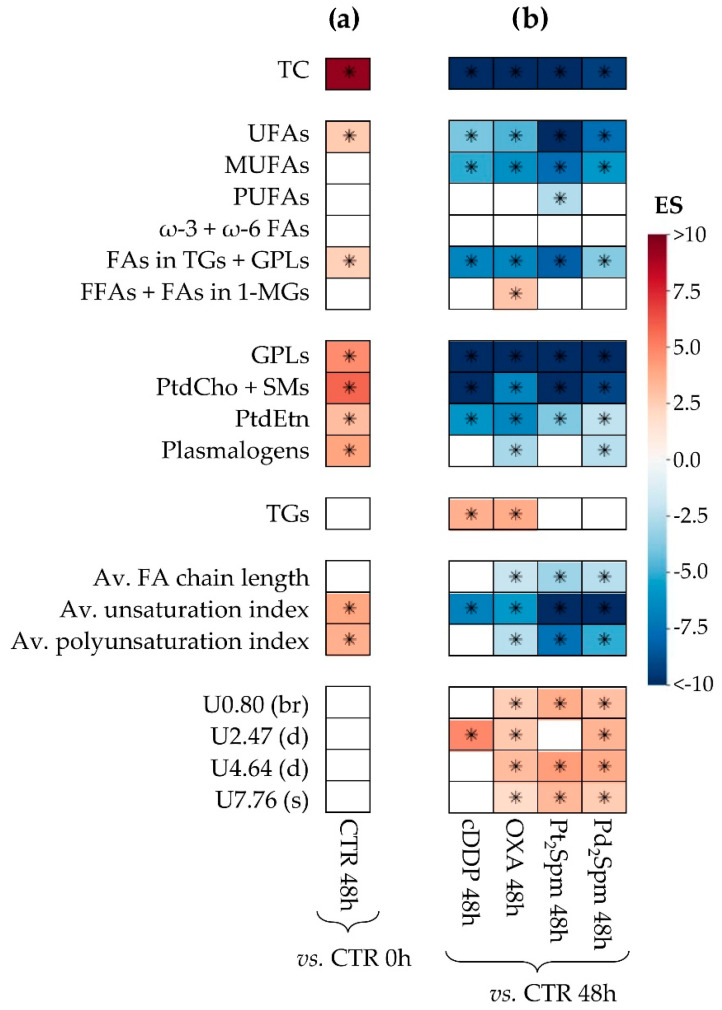
Heatmap showing statistically relevant variations found in different MG-63 lipid families (cholesterol, fatty acids (FAs), phospholipids and triglycerides), as well as in average FA chain length, unsaturation index, and polyunsaturation index, and some still unassigned resonances, Uδ (multiplicity/characteristics), under the following conditions: (**a**) before and after 48 h of proliferation and no drug treatment (CTR 0 h and CTR 48 h, respectively), and (**b**) upon treatment with cDDP, OXA, Pt_2_Spm or Pd_2_Spm for 48 h, compared to CTR 48 h. The color scale varies from minimum (dark blue) to maximum (dark red) effect size (ES) values [41]. *: Wilcoxon Rank-sum test *p*-value < 0.05, shown only in cases where spectral confirmation was observed. Abbreviations as defined in the caption of Figure 2.

**Figure 5:**
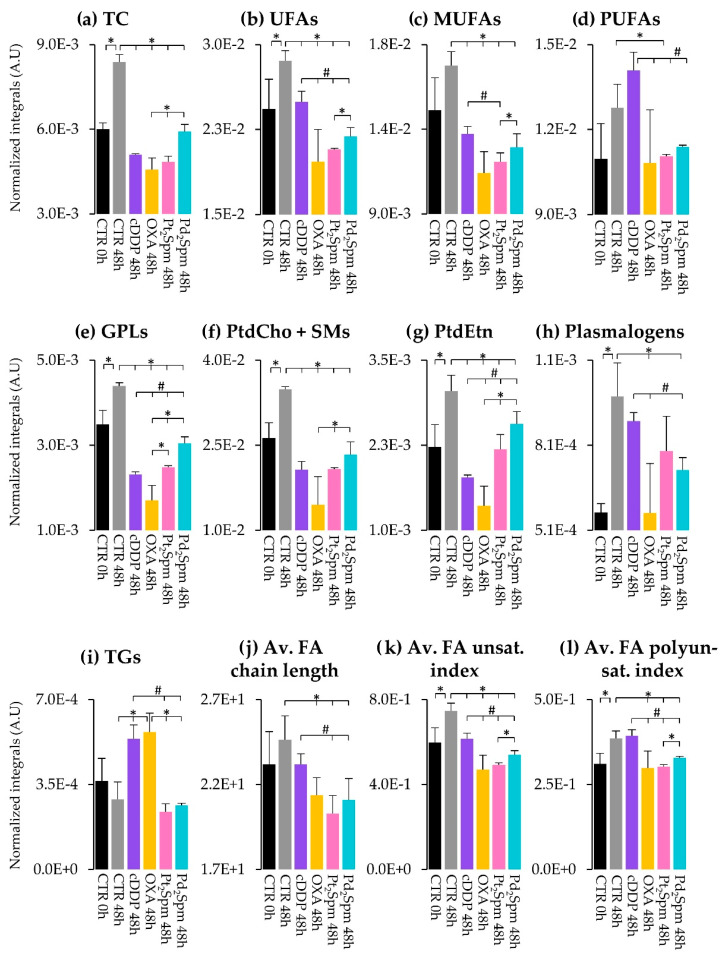
Lipidic levels of MG-63 cells before (CTR 0 h) and after 48 h of proliferation (CTR 48 h) or exposure to cDDP (purple), OXA (yellow), Pt_2_Spm (pink), and Pd_2_Spm (turquoise). Normalized integrals of lipids are shown, including (**a**) total cholesterol, (**b**–**d**) fatty acids (FAs), (**e**–**h**) phospholipids and (**i**) triglycerides. Average (**j**) FA chain length, (**k**) unsaturation index, and (**l**) polyunsaturation index were calculated according to [42]. Abbreviations as defined in the caption of Figure 2. Symbols above branched lines refer to comparisons between groups: *, Wilcoxon rank-sum test *p-*value < 0.05 (shown when also confirmed by visual inspection); #, relevant variations determined based on spectral confirmation, applicable when comparing cDDP (n = 2) with other small-sized groups (n ≤ 4).

Peak integration and univariate statistical analysis of lipid variations, expressed as effect size (ES) (Figure 4 and Table 1) and normalized integrals (Figure 5), confirm that tumor cell proliferation alone (controls at 0 and 48 h) is indeed accompanied by significant increases in (i) TC (Figure 5a), (ii) UFAs (which comprise MUFAs and PUFAs, which show increasing tendencies, Figure 5b–d), (iii) FAs incorporated in TGs and GPLs (Figure 4a) and (iv) several phospholipids, namely GPLs, PtdCho and SMs, PtdEtn and Pls (Figure 4a and Figure 5e–h). All the changes above had indeed been visually observed in the spectra (Figure 2) prior to statistical analysis. Control samples at 48 h show a qualitative decrease in TGs, i.e., without statistical relevance (Figure 5i), but possibly suggesting that higher cell proliferation at 48 h may occur partially at the expense of TG storage lipids. In addition, the noted increase in phospholipids is an expected response to the need for increased membrane synthesis, where increased UFAs and cholesterol (particularly in its free form [38,39]) may also be required to determine/maintain membrane fluidity properties, although cholesterol will certainly play many additional roles in proliferating cells [40]. Indeed, increased average FA unsaturation and polyunsaturation degrees (Figure 4a and Figure 5k,l) are consistent with some extension of increased membrane fluidity accompanying proliferation (despite the small tendency for average longer FA chain lengths, which may not only relate to membrane FAs but also to mostly saturated FAs, which integrate storage TGs).

#### 2.2.3. Lipidic Changes in MG-63 Cells Treated with Pt(II) Complexes cDDP, OXA and Pt_2_Spm (Same Metal, Distinct Ligands)

The impact of the three Pt(II) chelates on total cholesterol is similar, all agents inducing a marked decrease, in relation to control cells at the same time point (48 h) (Figure 5a). Unfortunately, no definite changes could be detected in either FC or EC due to the overlap and specific relative intensities of the corresponding resonances (EC appearing as a shoulder of the FC peak, which hindered reliable integration). It is interesting, however, to note the comparable TC decreases for all Pt(II) drugs, which indicates that, whatever roles are played by cholesterol (membrane fluidity and other bioactive roles), TC is independent of the ligand in Pt(II) chelates, regarding their use in treated MG-63 cells.

In terms of unsaturated FAs, a similar behavior is seen for total unsaturated FAs (or UFAs) and MUFAs, with all Pt(II) drugs decreasing the levels of these species, compared to the controls at 48 h, although such decreases are less marked for cDDP than for OXA and Pt_2_Spm, which reach comparable levels (Figure 5b,c). This generalized decrease in UFAs and MUFAs in treated cells may reflect the expected corresponding lower proliferation rates. A distinct effect is noted for PUFAs, the levels of which are left unchanged by cDDP exposure, whereas Pt_2_Spm and OXA (the latter displaying high variability) decrease them markedly, apparently to comparable extents (Figure 5d). This is reflected in the average FA unsaturation and polyunsaturation degrees (Figure 5k,l), the former following the same trend as UFAs and MUFAs, whereas the latter is not changed by cDDP (compared to the controls at 48 h) but decreases for both OXA and Pt_2_Spm. We hypothesize that unsaturated FAs will mainly integrate cell membranes and determine their fluidity and that the lesser cDDP-induced decrease in MUFAs and persistently high levels of PUFAs suggest that the cell membranes of cDDP-exposed cells exhibit higher fluidity, compared to OXA- or Pt_2_Spm-treated cells. The latter cells are, thus, expected to be characterized by more rigid, perhaps more resistant, cell membranes. We postulate that the possibly more fluid membranes upon cDDP treatment may be related to the drug´s lower IC_50_ (30 µM), compared to OXA (100 µM) and Pt_2_Spm (240 µM) [17], possibly increasing membrane permeability to the drug.

Membrane metabolism is, naturally, expected to be reflected on the phospholipids profile. It is interesting to note that all quantifiable phospholipid resonances (GPLs, PtdCho + SMs, PtdEtn and Pls, Figure 5e–h) show lower values for all Pt(II) agents, compared to the controls at 48 h, with OXA treatment apparently inducing the lowest values (although with high variability) compared to both cDDP and Pt_2_Spm. In the polar extracts of the same cells [18], increases in free choline and glycerophosphocholine (GPC) were associated with cDDP, OXA, and Pt_2_Spm action on MG-63 cells, with phosphocholine (PC) exhibiting a drug-dependent behavior (not changing with OXA and only increasing for Pt_2_Spm at 72 h). This was then interpreted as evidence of membrane degradation (although PC seemed to exhibit a slightly specific behavior), which is consistent with the general phospholipids decrease reported here. 

In relation to storage lipids, as viewed by TGs, it is interesting to note the marked increases in cDDP- and OXA-treated cells, in relation to the controls (Figure 5i), whereas TG levels upon Pt_2_Spm treatment remain much lower and comparable to those in controls. Increases in MG-63 cell lipids with cDDP treatment, as viewed by direct pellet analysis through high-resolution magic angle spinning (HRMAS) NMR, have been reported before [16,30,31] and mostly interpreted as increased cytosolic lipid droplets (although also suggested to have possible contributions from TG microdomains embedded in the plasma membrane) [43]. Lipid increase has also been seen in tumor cells and in radiation-exposed MG-63 cells [35] and is generally suggested to reflect cell apoptosis mechanisms. Hence, while an increase in FA content (here viewed in the form of TGs) with treatment time is expected for MG-63 cells upon cDDP, it is interesting that it occurs to high extents in both cDDP- and OXA-treated cells, whereas it is not observed in Pt_2_Spm-treated cells. It is indeed possible that the extension of apoptosis is lower in the latter situation, in agreement with the highest IC_50_ (240 µM) seen for Pt_2_Spm, within all Pt(II) chelates. Although apoptosis could not be quantified for the samples studied here, we hypothesize that higher degrees of apoptosis may explain the high TG values for cDDP and OXA, reflecting comparable apoptotic behavior despite the differences in IC_50_ values (30 and 100 µM, respectively). Interestingly, the change to a dinuclear Pt(II) chelate with spermine does not impact on TG levels, probably as a result of less/no apoptosis. HRMAS studies of cell pellets have also reported an increase in the overall FA average unsaturation degree and chain length for cDDP-treated MG-63 cells [30], also interpreted as evidence of apoptosis. Such changes oppose those observed in the cell extracts studied here (tendencies for a lower FA average chain length and unsaturation degree for drug-treated cells, compared to the controls), an observation that may be explained by the fact that HRMAS NMR records the signals from the more mobile lipids (and predictably not their total amount), whereas the NMR of liquid extracts is expected to account for all lipids present in the sample. This may be seen as one of the advantages of the extract analysis compared to the HRMAS direct analysis (depending on the aim of the study, naturally), adding to the fact that the NMR of extracts enables higher resolution to be achieved and, hence, better differentiation between distinct lipid families. It is interesting that the previously reported metabolomic analysis of the polar extracts of these cells suggested higher tricarboxylic acid (TCA) activity (and a less active Warburg effect) for both cDDP and OXA-treated cells, compared to Pt_2_Spm [18]. It is possible that this switch for a more energetically favorable phenotype, based on an enhanced oxidative phosphorylation (OXPHOS) profile, may be feeding enhanced de novo lipid biosynthesis, here expressed by higher TG levels.

#### 2.2.4. Lipidic Changes in MG-63 Cells Treated with Pt_2_Spm and Pd_2_Spm (Same Ligand, Distinct Metal Centers)

Generally, Pd_2_Spm (with an IC_50_ value of 24 µM, comparable to that of cDDP, 30 µM) induces a significantly more different lipid profile in MG-63 cells than its Pt(II) analog. The impact of Pd_2_Spm, compared to Pt_2_Spm, is characterized by generally higher levels, namely of TC, UFAs, MUFAs, PUFAs (tendency only), GPLs, PtdCho + SMs, PtdEtn, as well as average unsaturation and polyunsaturation degrees (mostly reflecting the higher levels of MUFAs (Figure 5a–g,k,l)). In fact, all the above-mentioned lipid species and ratios are decreased, compared to the controls at 48 h, in Pd_2_Spm-treated cells, but not as much as seen for Pt_2_Spm (and OXA). This behavior generally approaches Pd_2_Spm-induced levels of TC, several unsaturated FAs, and phospholipids, to those characterizing cDDP-treated cells. We hypothesize that the relative increases in TC and, particularly, in unsaturated FAs (including unsaturation and polyunsaturation degrees), indicate that Pd_2_Spm-treated cells exhibit higher membrane fluidity, compared to Pt_2_Spm and OXA, and similarly to cDDP, thus probably justifying the higher and similar cytotoxicity of both Pd_2_Spm and cDDP (in terms of drug facilitated access to the intracellular environment). In addition, the similar tendency for the most abundant phospholipids (higher than Pt_2_Spm and OXA, and approaching or similar to cDDP) initially suggests similar degrees of membrane degradation for both Pd_2_Spm and cDDP. However, PC levels in polar extracts [18] were significantly higher in Pd_2_Spm-treated cells (ES 19 and 7 at 48 and 72 h, respectively) than in Pt_2_Spm-treated cells (only change noted at 72 h, with ES 2), with values tending (but still lower) to those characterizing cDDP-treated cells (ES 7 and 3, at 48 and 72 h). We suggest, therefore, that the marked PC increases upon Pd_2_Spm treatment may express a preferential use of PC as a precursor for sustained phospholipid biosynthesis in surviving MG-63 cells, particularly upon exposure to Pd_2_Spm (as supported by the relatively higher GPL, PtdCho + SM and PtdEtn levels observed for both Pd_2_Spm and cDDP). Phospholipid metabolism seems, therefore, to reflect a fine equilibrium between degradation and biosynthesis, possibly resulting in the inclusion of more unsaturated FAs, thus leading to more fluid membranes and more drug-accessible cells.

Notably, Pd_2_Spm- and Pt_2_Spm-treated MG-63 cells show equivalent low levels of TGs, markedly lower than those characterizing the effects of cDDP and OXA (Figure 5h,i). Both Pt_2_Spm and Pd_2_Spm-treated cells exhibited lower TCA cycle activity, compared to cDDP [18], supporting the idea that enhanced OXPHOS may energetically support enhanced TG synthesis. Above, these lipids were suggested to relate to the degree/occurrence of cell apoptosis and, hence, the unchanged TG levels (compared to the controls at 48 h) characterizing cells treated with both spermine chelates seem to indicate the absence of apoptosis in both cases. Indeed, no apoptosis was detected in a previous HRMAS NMR study of Pd_2_Spm treatment of MG-63 cell pellets, contrary to the high apoptotic behavior of cDDP-treated cells [16] and this is in agreement with the observations reported here, for MG-63 cell lipidic extracts. 

We, therefore, suggest that the mechanism of action of Pd_2_Spm on MG-63 cells, as viewed by the lipidome adaptations, does not involve a high extent of apoptosis as cDDP does, but rather an adaptation of cell membrane composition and fluidity (strongly supported by high levels of PC), to facilitate the entrance of Pd_2_Spm into the cells. Once in an intracellular environment of seemingly non-apoptotic MG-63 cells (or, at least, less apoptotic than with cDDP), Pd_2_Spm has been reported to give rise to significantly distinct amino acids and nucleotides responses (lower TCA and OXPHOS activation, and lesser reversal of the Warburg effect [18]), which previously suggested a different mechanism of action to the highly apoptotic induction of cDDP. The present results add that the Pd_2_Spm action seems indeed to be accompanied by less/absence apoptosis as well as an active role of PC in membrane biosynthesis/repair processes, possibly resulting in higher membrane fluidity and, thus, easier drug entrance into the cells.

## 3. Materials and Methods

### 3.1. Chemicals and Solutions

Cisplatin (*cis*-diamminedichloro platinum(II), cDDP > 99.9%), minimum essential medium (MEM), non-essential amino acids (NEAA), oxaliplatin (OXA), penicillin/streptomycin 100× solution, phosphate-buffered saline (PBS), potassium tetrachloroplatinate(II) (K_2_PtCl_4_, >99.9%), potassium tetrachloropalladate(II) (K_2_PdCl_4_, >99.9%), sodium pyruvate (100 mM), spermine (*N*,*N*′*-*bis(3-aminopropyl)-1,4- diaminobutane, free base, Spm), Trypan blue (0.4% *w*/*v*), trypsin-EDTA (1×), as well as solvents, inorganic salts, and acids (analytical grade) were purchased from Sigma-Aldrich Chemical S.A. (Sintra, Portugal). Fetal bovine serum (FBS) was obtained from Gibco-Life Technologies (Grand Island, NY, USA). Pt_2_Spm and Pd_2_Spm were synthesized according to published procedures [44] optimized by the authors [45]. Briefly, 2 mmol of K_2_PdCl_4_ or K_2_PtCl_4_ were dissolved in a small amount of water, and 1 mmol of Spm (aqueous solution) was added dropwise, under stirring. After 24 h, the resulting powder was filtered and washed with acetone. For drug administration, initial stock solutions of cDDP (960 µM), OXA (1 mM), Pt_2_Spm (2.4 mM), and Pd_2_Spm (960 µM) were prepared in: PBS, for cDDP and OXA; and PBS with DMSO 20% (*v*/*v*), for Pt_2_Spm and Pd_2_Spm. All solutions were filtered (0.20 µm syringe filters) and stored at −20 °C.

### 3.2. Cell Culture

The human osteosarcoma MG-63 cell line was obtained from ECACC (European Collection of Authenticated Cell Cultures, UK). Cells were grown as monolayers under a humidified atmosphere with 5% CO_2_ at 37 °C, in MEM culture medium, supplemented with 10% (*v*/*v*) heat-inactivated FBS, 1% (*v*/*v*) sodium pyruvate, 1% (*v*/*v*) NEAA and antibiotics (penicillin/streptomycin 10×).

### 3.3. Sample Preparation

MG-63 cell cultures were established in 100 mm diameter Petri dishes at a density of 3.0 × 10^4^ cells/cm^2^ in order to provide a sufficiently high cell density for NMR analysis, upon well pooling when necessary. After waiting 24 h for cells to adhere, the experiment was started (t = 0 h) by adding the IC_50_ concentration of each drug (previously determined [17]): 30 µM cDDP, 100 µM OXA, 240 µM Pt_2_Spm and 24 µM Pd_2_Spm. According to the population doubling time for MG-63 cells (24 h), the 48 h time-point after drug addition was chosen for sample collection and compared with 0 h. Cells were harvested by trypsinization, washed twice with PBS, centrifuged (at 300× *g*, 5 min), and counted by the Trypan blue exclusion assay. These cell pellets were then snap-frozen in liquid nitrogen and stored at −80 °C until cell extraction for NMR analysis. Two independent experiments with triplicates for each condition (time-point and drug) were set up; however, some samples had to be discarded due to technical reasons and final group sizes were as follows: controls 0 h n = 3, controls 48 h n = 6, cDDP 48 h n = 2, OXA 48 h n = 4, Pt_2_Spm 48 h n = 4 and Pd_2_Spm 48 h n = 3.

### 3.4. Cell Extraction

Intracellular lipidic metabolites were extracted using a dual-phase extraction, with methanol/chloroform/water, as described elsewhere [46,47]. Briefly, 650 µL of cold methanol 80% was added to cell pellets, quickly vortexed (5 min), transferred into microcentrifuge tubes containing 0.5 mm glass beads, to aid cell breakage, and vortexed for 5 min. Cold chloroform (260 μL) was added, and samples were vortexed, followed by the addition of cold chloroform (260 μL) and Milli-Q water (220 μL), and vortexed again. Samples were then left to rest in ice for 10 min, followed by centrifugation (2000× *g*, 15 min), and the lower (lipidic) phase was transferred into new vials, dried under nitrogen, and stored at −80 °C until NMR analysis.

### 3.5. NMR Spectroscopy

The NMR spectra of lipophilic extracts were carried out after reconstitution of the dry extracts in 600 μL of deuterated chloroform (99.8% deuterium) with 0.03% tetramethylsilane (TMS) for chemical shift referencing. After vortexing, 550 μL of each sample was transferred into 5 mm NMR tubes. NMR spectra were acquired on a Bruker Avance DRX-500 spectrometer operating at 500.13 MHz for ^1^H observation, at 298 K, using a 5 mm probe. Standard 1D ^1^H NMR spectra were recorded using the “zg” pulse sequence (Bruker library), with a 7002.801 Hz spectral width, 32 k data points, a 2 s recycle delay (d1), a 2.34 s acquisition time (hence giving a total of 4.34 s of relaxation delay) and 512 transients. Prior to Fourier transformation, free-induction decays were zero-filled to 65 k points and multiplied by a 0.3 Hz exponential function. 1D ^1^H NMR spectra were manually phased, baseline-corrected, and chemical shift referenced to the TMS signal (δ 0.00). 2D ^1^H/^1^H total correlation (TOCSY), ^1^H/^13^C heteronuclear single quantum correlation (HSQC), and *J*-resolved (*J*-res) spectra were acquired for selected samples to aid spectral assignment, although, due to their lower resolution (compared to polar samples), peak assignment relied more on comparison with existing literature [48,49,50,51], spectral databases, such as the Bruker BIOREFCODE database and the human metabolome database (HMDB) [52], and spiking experiments (namely to verify the presence of phosphatidylcholine (PtdCho), phosphatidylethanolamine (PtdEtn), lysophosphatidylcholine (LysoPtdCho), sphingomyelins (SMs), mono-, di- and triglycerides (MGs, DGs, TGs), free and esterified cholesterol (Chol., EChol.), oleic acid (OA), arachidonic acid (AHA) and docosahexaenoic acid (DHA)).

### 3.6. Data Processing and Statistics

The full resolution 1D ^1^H NMR spectra were converted into matrices (AMIX-viewer 3.9.14, Bruker BioSpin, Rheinstetten, Germany), after exclusion of the chloroform and corresponding satellites region (δ 6.90–7.65), and water region (δ 1.43–1.79). Spectra were aligned using recursive segment-wise peak alignment (MATLAB 8.3.0, The MathWorks, Inc., Natick, MA, USA), to minimize chemical shift variations [53], and normalized to total spectral area, to account for differences in cell numbers. After unit variance (UV) scaling (SIMCA-P 11.5; Umetrics, Umeå, Sweden) [54], multivariate analysis was carried out using PCA, an unsupervised method used to detect intrinsic clusters and outliers within the data set, and PLS-DA, a supervised method used to maximize class discrimination. PLS-DA models were considered statistically robust for predictive power (Q^2^) values ≥ 0.50. For selected models, PLS-DA loadings were retrieved and colored by VIP for analysis. Lipid changes suggested by loadings analysis were validated by integration, as follows. A representative peak of each spin system was integrated (AMIX 3.9.5, Bruker BioSpin, Rheinstetten, Germany), normalized to total spectral area, and variations assessed by univariate analysis (Wilcoxon rank-sum test) [55] (Python 3.9, Python Software Foundation, Fredericksburg, VA, USA). Statistical significant metabolite alterations (*p*-values < 0.05, absolute value of effect size (ES) > 0.5, and ES error < 80% [41]) were expressed in heatmaps colored as a function of ES.

## 4. Conclusions

This NMR metabolomics study reports distinct lipophilic responses of MG-63 cells when treated with several Pt(II)/Pd(II) chelates, including conventional drugs, cDDP and OXA, and potential spermine drugs, Pt_2_Spm and Pd_2_Spm. Although not as often used as MS methods to characterize lipids, NMR measures the response of several lipid families simultaneously, namely cholesterol, FAs, and related characteristics (average chain length and unsaturation/polyunsaturation degrees), several phospholipids, and TGs. Proliferation alone (control conditions) was seen to require higher contents of phospholipids, as membrane building blocks, while a slight FA unsaturation enhancement suggested a small increase in membrane fluidity. Regarding cell exposure, it became clear that the three Pt(II) chelates induced distinct lipidome responses, namely, the levels of PUFAs were kept high by cDDP, while they decreased with OXA or Pt_2_Spm. Supposing that PUFAs will mainly integrate membrane phospholipids, it is possible that cell membranes become more fluid upon cDDP treatment, increasing cell permeability and hence contributing to higher drug cytotoxicity (IC_50_ of 30 µM for cDDP, compared to 100 µM and 240 µM, for OXA and Pt_2_Spm, respectively). Concomitantly, all quantifiable phospholipids (GPLs, PtdCho + SMs, PtdEtn, and Pls) were decreased compared to the controls, suggesting membrane degradation, in agreement with previously reported high contents of polar degradation products (mainly choline and GPC). Remarkably, cDDP and OXA significantly increased TG levels, while Pt_2_Spm left them unchanged relative to the controls. We hypothesize that this reflects enhanced apoptotic behavior induced by cDDP and OXA, but not by the dinuclear Pt_2_Spm chelate. Given this hypothesis, a limitation of this work is that apoptosis quantification could not be carried out, thus remaining the subject of ongoing work. We further suggest that a previously discussed switch from lactate synthesis toward aerobic oxidative phosphorylation in MG-63 cells exposed to cDDP or OXA may serve as energy support for enhanced TG biosynthesis. 

Comparison of both spermine chelates is most interesting, as the Pd(II) agent has revealed promising cytotoxicity properties in several earlier reports. The lipidome behaved quite distinctly as a result of Pt replacement by Pd, namely exhibiting phospholipid levels approaching those in cDDP-treated cells (i.e., higher than in OXA- or Pt_2_Spm-exposed cells). We propose that, for cDDP and Pd_2_Spm, phospholipid metabolism reflects a different point of degradation/biosynthesis equilibrium, with a higher contribution of de novo biosynthesis (possibly supported by PC as a preferential precursor, based on reported polar metabolome adaptations), to incorporate more unsaturated FAs (also seen increased in Pd_2_Spm-exposed cells, compared to OXA and Pt_2_Spm). This would lead to more fluid membranes and more drug-accessible cells. Notably, Pd_2_Spm- and Pt_2_Spm-treated MG-63 cells show equivalent (low) levels of TGs, which suggests the absence (or low extent) of apoptosis in the corresponding treated cells. Hence, our results suggest that the mechanism of action of Pd_2_Spm on MG-63 cells does not seem to involve apoptosis, as cDDP, but rather an adaptation of cell membrane composition and fluidity to facilitate the entrance of Pd_2_Spm into the cells (such an effect is also suggested for cDDP but in tandem with apoptosis induction). This is proposed as a possible mechanism through which Pd_2_Spm exerts its high cytotoxicity toward MG-63 cells (comparably to cDDP), thus supporting the promise of the Pd(II) chelate as a possible Pt(II) substitute in clinical therapy.

## Figures and Tables

**Table 1:** Statistically significant lipid variations (|ES| > 0.50, ES error < 80%, Wilcoxon rank-sum *p*-value < 0.05) of MG-63 cells after 48 h exposure to cDDP, OXA, Pt_2_Spm, and Pd_2_Spm, compared to the controls (untreated cells in the same 48 h period). Effect size (ES) values and corresponding errors were calculated according to reference [41]. All differences presented were confirmed by visual inspection of the spectra. Statistical significance is only indicated qualitatively (*p*-values < 0.05), i.e., without specifying *p*-values, due to the low sample numbers in each group (particularly in cDDP-treated cells for which n = 2, due to technical reasons).

Compound	cDDP 48 h		OXA 48 h		Pt_2_Spm 48 h		Pd_2_Spm 48 h	
	ES (Error (%))	*p-*Value	ES (Error (%))	*p-*Value	ES (Error (%))	*p-*Value	ES (Error (%))	*p-*Value
TC	−13.3 (50.4)	<0.05	−11.5 (45.2)	<0.05	−14.4 (44.7)	<0.05	−9.3 (48.5)	<0.05
UFAs	−4.0 (63.5)	<0.05	−4.7 (51.4)	<0.05	−10.8 (45.4)	<0.05	−7.6 (49.6)	<0.05
MUFAs	−5.1 (58.2)	<0.05	−6.2 (48.3)	<0.05	−7.7 (46.8)	<0.05	−5.8 (52.0)	<0.05
PUFAs	-	-	-	-	−2.6 (65.7)	<0.05	-	-
FAs in TGs + GPLs	−6.7 (54.5)	<0.05	−6.6 (47.9)	<0.05	−8.2 (46.5)	<0.05	−3.7 (59.4)	<0.05
FFAs + FAs in 1-MGs	-	-	2.9 (62.3)	<0.05	-	-	-	-
GPLs	−27.3 (49.4)	<0.05	−12 (45.1)	<0.05	−29.1 (44.0)	<0.05	−12.9 (47.4)	<0.05
PtdCho + SMs	−20.4 (49.6)	<0.05	−6.6 (47.8)	<0.05	−37.5 (44.0)	<0.05	−9 (48.7)	<0.05
PtdEtn	−5.9 (55.9)	<0.05	−6.6 (47.8)	<0.05	−3.8 (55.2)	<0.05	−2.2 (78.0)	<0.05
Pls	-	-	−2.9 (62.1)	<0.05	-	-	−2.5 (72.2)	<0.05
TGs	3.6 (66.1)	<0.05	3.8 (55.2)	<0.05	-	-	-	-
Av. FA chain length	-	-	−2.1 (74.7)	<0.05	−3.1 (60.3)	<0.05	−2.5 (72.5)	<0.05
Av. FA unsat. index	−6.8 (54.3)	<0.05	−5.8 (48.9)	<0.05	−20.7 (44.2)	<0.05	−13.9 (47.3)	<0.05
Av. FA polyunsat. index	-	-	−2.5 (67.5)	<0.05	−7.4 (47.0)	<0.05	−5 (53.8)	<0.05
Uδ0.80 (br)	-	-	2.4 (68.0)	<0.05	3.7 (55.5)	<0.05	3 (65.0)	<0.05
Uδ2.47 (d)	4.9 (58.9)	<0.05	2.7 (64.1)	<0.05	-	-	3.4 (61.3)	<0.05
Uδ4.64 (d)	-	-	3.2 (59.2)	<0.05	4.3 (52.9)	<0.05	3.8 (58.9)	<0.05
Uδ7.76 (s)	-	-	1.9 (79.9)	<0.05	3.3 (58.3)	<0.05	2.6 (71.1)	<0.05

## Data Availability

The data presented in this study will be openly available in the Metabolomics Workbench: An international repository for metabolomics data and metadata, metabolite standards, protocols, tutorials and training, and analysis tools (2016), website: https://www.metabolomicsworkbench.org (accessed on 12 December 2023). An identification number and link will be available in due time.

## Supplementary Materials

The following supporting information can be downloaded at https://www.mdpi.com/article/10.3390/ijms242417556/s1.
