# Supplementary material for: Impact of Conventional and Potential New Metal-Based Drugs on Lipid Metabolism in Osteosarcoma MG-63 Cells
Source: Int J Mol Sci. 2023 Dec 16;24(24):17556. doi: 10.3390/ijms242417556 (PMC10743680; doi:10.3390/ijms242417556)
Supplement: Supplementary file 1 [file ijms-24-17556-s001.zip › ijms-2748937-supplementary.pdf]

International Journal of Molecular Sciences

SUPPORTING INFORMATION

Impact of Conventional and Potential New Metal-Based Drugs on Lipid Metabolism in Osteosarcoma MG-63 Cells

Daniela S. C. Bispo [1,†], Marlene Correia [1,†], Tatiana J. Carneiro [1,2], Ana S. Martins [1,2], Aliana A. N. Reis [1], Ana L. M. Batista de Carvalho [2], Maria P. M. Marques [2,3] and Ana M. Gil [1,*]

[1] Department of Chemistry, CICECO—Aveiro Institute of Materials (CICECO/UA), University of Aveiro, Campus Universitário de Santiago, 3810-193 Aveiro, Portugal; d.bispo@ua.pt (D.S.C.B.); marlene24@ua.pt (M.C.); tatiana.joao@ua.pt (T.J.C.); ascm@ua.pt (A.S.M.); aliananosolini@ua.pt (A.A.N.R.)
[2] Unidade de I&D Química-Física Molecular, Department of Chemistry, University of Coimbra, Rua Larga, 300-535 Coimbra, Portugal; almbc@uc.pt (A.L.M.B.d.C.); pmc@ci.uc.pt (M.P.M.M.)
[3] Department of Life Sciences, Faculty of Science and Technology, University of Coimbra, Calçada Martim de Freitas, 3000-456 Coimbra, Portugal
* Correspondence: agil@ua.pt; Tel.: +351-234370707
† These authors contributed equally to the paper.

This Supporting Information section includes:

Table S1. ^{1}H NMR assignment (500 MHz) of lipidic metabolites identified in MG-63 cellular extracts, listed by increasing chemical shift value. Metabolite abbreviations: 1-MGs, 1-monoacylglycerides; EC, esterified cholesterol; FAs, fatty acids; FC, free cholesterol; FFAs, free fatty acids; GPLs, glycerophospholipids; MUFAs, monounsaturated fatty acids; PtdCho, phosphatidylcholine; PtdEtn, phosphatidylethanolamine; PUFAs, polyunsaturated fatty acids; SMs, sphingomyelins; TC, total cholesterol; TGs, triglycerides; UFAs, unsaturated fatty acids; Multiplicity: s, singlet; br, broad signal; t, triplet; m, multiplet; d: doublet, dd, doublet of doublets; ddd, doublet of doublets of doublets. [a], Specific signals arising from DHA, EPA, and AA could not be identified; [†], tentative assignment

Figure S1. PCA score scatter plots obtained for ^{1}H NMR spectra of lipidic extracts of MG-63 cells after 48 hours of exposure time to **a)** 30 μM cDDP, **b)** 100 μM OXA, **c)** 240 μM Pt_2Spm, and **d)** 24 μM Pd_2Spm, compared to controls at 48 hours; **e)** Pt_2Spm is compared to Pd_2Spm, and **f)** the three Pt(II) complexes, cDDP, OXA, and Pt_2Spm.

Table S1. ^{1}H NMR (500 MHz) assignment of lipidic metabolites identified in MG-63 cellular extracts, listed by increasing chemical shift value. Metabolite abbreviations: 1-MGs, 1-monoacylglycerides; EC, esterified cholesterol; FAs, fatty acids; FC, free cholesterol; FFAs, free fatty acids; GPLs, glycerophospholipids; MUFAs, monounsaturated fatty acids; PtdCho, phosphatidylcholine; PtdEtn, phosphatidylethanolamine; PUFAs, polyunsaturated fatty acids; SMs, sphingomyelins; TC, total cholesterol; TGs, triglycerides; UFAs, unsaturated fatty acids; Multiplicity: s, singlet; br, broad signal; t, triplet; m, multiplet; d: doublet, dd, doublet of doublets; ddd, doublet of doublets of doublets. [a], Specific signals arising from DHA, EPA, and AA could not be identified; †, tentative assignment.

**Metabolite**		**δ ^{1}H in ppm (multiplicity, assignment)**
Cholesterol	Total	0.68 (s, 18-C$\underline{\mathbf{H}}_3$)
	Total	0.86 (d, 26-C$\underline{\mathbf{H}}_3$)
	Total	0.87 (d, 27-C$\underline{\mathbf{H}}_3$)
	Total	0.91 (d, 21-C$\underline{\mathbf{H}}_3$)
	Free	1.01 (s, 19-C$\underline{\mathbf{H}}_3$)
	Esterified	1.02 (s, 19-C$\underline{\mathbf{H}}_3$)
	Total	1.12 (m, multiple cholesterol protons)
	Total	1.48 (m, multiple cholesterol protons)
	Total	1.84 (m, multiple cholesterol protons)
	Total	1.96 (br, 7–C$\underline{\mathbf{H}}_2$/8–C$\underline{\mathbf{H}}$)
	Total	2.23 (br, 4-C$\underline{\mathbf{H}}_2$)
	Free	3.53 (br, 3-C$\underline{\mathbf{H}}$)
	Esterified	4.61 (m, 3-C$\underline{\mathbf{H}}$)
	Total	5.32 (br, 6-C$\underline{\mathbf{H}}$)
Fatty acids (FAs)	All FAs (except ω-3)	0.88 (t, C$\underline{\mathbf{H}}_3$)
	ω-3 FAs	0.97 (t, C$\underline{\mathbf{H}}_3$)
	All FAs	1.27 (m, (C$\underline{\mathbf{H}}_2$)$_n$)
	All FAs (except DHA, EPA, and AA[a])	1.60 (m, -C$\underline{\mathbf{H}}_2$-CH$_2$-CO-)
	All UFAs	1.98-2.08 (m, -CH$_2$-C$\underline{\mathbf{H}}_2$-CH=)
	MUFAs	2.01 (m, -CH$_2$-C$\underline{\mathbf{H}}_2$-CH=)
	ω-3 and ω-6 FAs (except AA and DHA[a])	2.06 (m, -CH$_2$-C$\underline{\mathbf{H}}_2$-CH=)
	FAs in TGs + GPLs + EC (except DHA[a])	2.29 (m, -C$\underline{\mathbf{H}}_2$-CO)
	FFAs + FAs in 1-MGs (except DHA[a])	2.35 (t, -C$\underline{\mathbf{H}}_2$-CO)
	LA (18:2, ω-6)	2.77 (t, =CH-C$\underline{\mathbf{H}}_2$-CH=)
	PUFAs (except LA)	2.82 (m, =CH-C$\underline{\mathbf{H}}_2$-CH=)
	UFAs	5.35 (m, -$\underline{\mathbf{H}}$C=C$\underline{\mathbf{H}}$-)
Glycerophospholipids (GPLs) and sphingomyelins (SMs)	PtdEtn	3.16 (br, N-C$\underline{\mathbf{H}}_2$ of ethanolamine)
	SMs	3.30 (s, -N$^+$(C$\underline{\mathbf{H}}_3$)$_3$)
	PtdCho	3.32 (s, -N$^+$(C$\underline{\mathbf{H}}_3$)$_3$ of choline)
	PtdCho + SMs	3.74 (br, N-C$\underline{\mathbf{H}}_2$ of choline)
	Plasmalogens †	3.85 (m, 1-C$\underline{\mathbf{H}}_2$ of glycerol)
	All GPLs	3.95 (m, PO-(3-C$\underline{\mathbf{H}}_2$) of glycerol)
	PtdEtn	4.07 (br, PO-C$\underline{\mathbf{H}}_2$ of ethanolamine)
	All GPLs	4.36 (m, 1-C$\underline{\mathbf{H}}_2$ of glycerol)
	Plasmalogens †	5.16 (m, 2-C$\underline{\mathbf{H}}_2$ of glycerol)
	All GPL	5.22 (m, 2-C$\underline{\mathbf{H}}$ of glycerol)
	SMs	5.68 (m, -CH$_2$-C$\underline{\mathbf{H}}$=CH-CHOH-)
	Plasmalogens	5.90 (d, O-C$\underline{\mathbf{H}}$=CH)
Glycerolipids	1-MGs	3.65 (ddd, 3-C$\underline{\mathbf{H}}_2$ of glycerol)
	TGs	4.15 (dd, 1-C$\underline{\mathbf{H}}_2$/3-C$\underline{\mathbf{H}}_2$ of glycerol)
	1-MGs	4.18 (ddd, 1-C$\underline{\mathbf{H}}_2$ of glycerol)
	TGs	4.29 (dd, 1-C$\underline{\mathbf{H}}_2$/3-C$\underline{\mathbf{H}}_2$ of glycerol)
	TGs	5.28 (m, 2-C$\underline{\mathbf{H}}$ of glycerol)

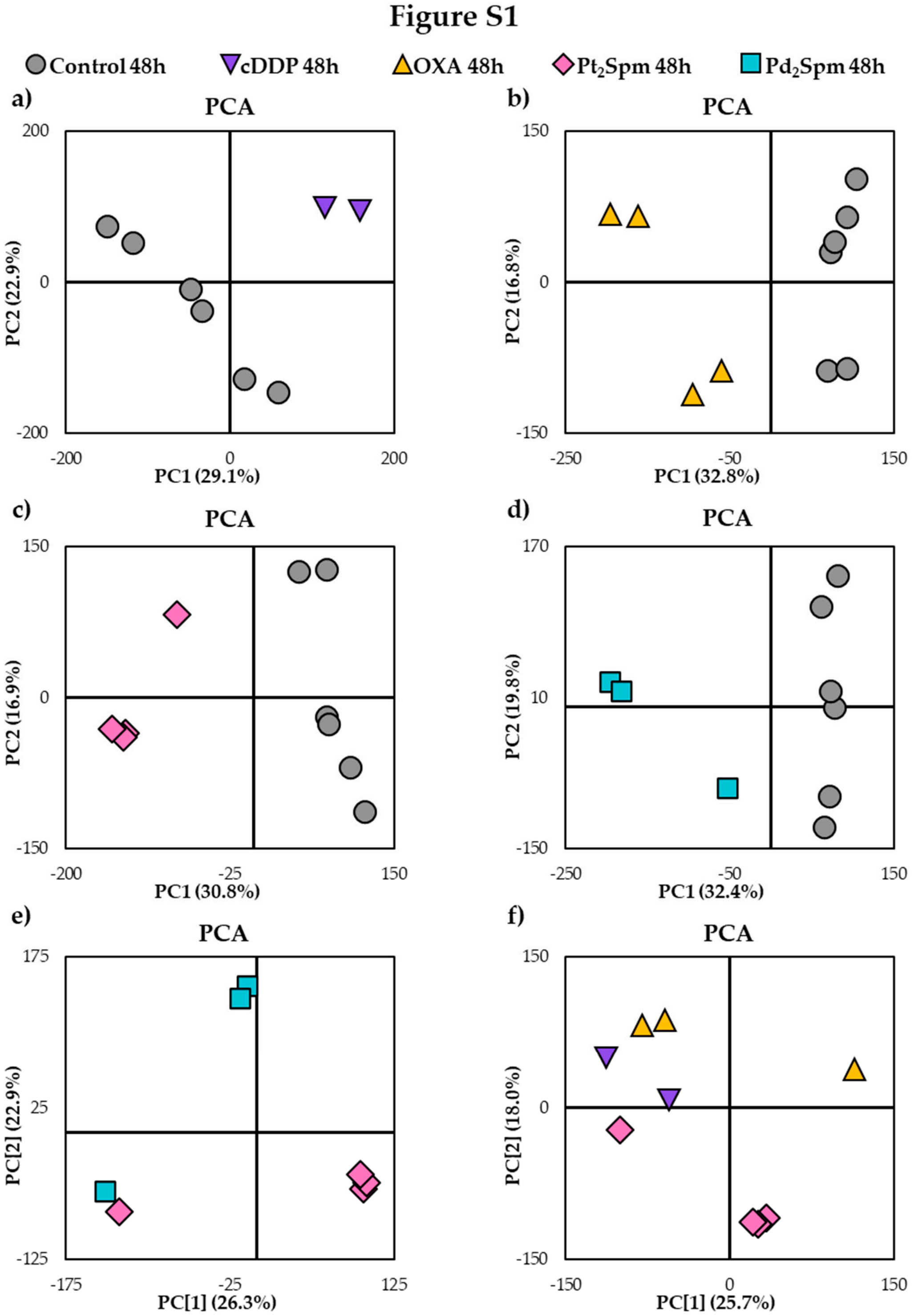

Figure S1. PCA score scatter plots obtained for ^{1}H NMR spectra of lipidic extracts of MG-63 cells (in $CDCl_3$) after 48 hours of exposure time to **a)** 30 μM cDDP, **b)** 100 μM OXA, **c)** 240 μM Pt_2Spm, and **d)** 24 μM Pd_2Spm, compared to controls at 48 hours; **e)** Pt_2Spm compared to Pd_2Spm, and **f)** the three Pt(II) complexes, cDDP, OXA, and Pt_2Spm.
